# Management strategies following implantation failure of euploid embryos

**DOI:** 10.1002/rmb2.12576

**Published:** 2024-04-08

**Authors:** Keiji Kuroda

**Affiliations:** ^1^ Center for Reproductive Medicine and Endoscopy Sugiyama Clinic Marunouchi Tokyo Japan; ^2^ Department of Obstetrics and Gynaecology Juntendo University Faculty of Medicine Tokyo Japan

**Keywords:** euploid embryo, implantation failure, in vitro fertilization, infertility, preimplantation genetic testing

## Abstract

**Background:**

Euploid blastocyst implantation failure may result from embryonic factors undetectable by preimplantation genetic testing for aneuploidy (PGT‐A); however, various nonembryonic factors can also intricately interfere with implantation. This review seeks to clarify evidence‐based testing and treatments for implantation failure after euploid embryo transfer.

**Methods:**

We conducted a review of the literature on implantation failure after euploid embryo transfer or multiple embryo transfer cycles, which mainly included systematic reviews and meta‐analyses.

**Results:**

The recommended tests for implantation failure include (1) hysteroscopy, (2) endometrial CD138 immunohistochemistry and bacterial culture, (3) serum 25‐hydroxyvitamin D_3_, and (4) thrombophilia screening. Based on diagnostic findings, the following treatments have been recommended: (1) antibiotics for chronic endometritis, (2) vitamin D replacement, (3) lifestyle modification, and (4) low‐dose aspirin starting from the postimplantation period for thrombophilia. Moreover, frozen–thawed single euploid blastocyst transfer using assisted hatching and hyaluronan‐enriched transfer medium may support embryo implantation.

**Conclusion:**

To ensure a successful pregnancy in subsequent embryo transfers, simple, inexpensive, and evidence‐based tests and treatments should be selected.

## Graphical abstract

Euploid blastocyst implantation failure result from embryonic factors undetectable by preimplantation genetic testing for aneuploidy or nonembryonic risk factors including intrauterine abnormalities, imbalance in maternal immunity, hydrosalpinx, and luteal phase deficiency. Hypothyroidism, thrombophilia, and lifestyle habits mainly increase the risk of pregnancy loss but not implantation failure. The cornerstone for the treatment of implantation failure is to identify and address the causes of the unsuccessful pregnancy.

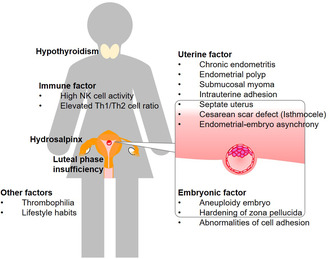

## 1 INTRODUCTION

Detection of a euploid embryo after expensive assisted reproductive technology (ART) treatment with preimplantation genetic testing for aneuploidy (PGT‐A) can lead patients to believe that they can finally become pregnant. Therefore, failure of a euploid embryo to implant can cause unimaginable levels of disappointment among patients. At embryo implantation, the uterine endometrium is decidualized by progesterone secreted from the corpus luteum, creating a period of embryo implantation known as the window of implantation (WOI). The decidualized endometrium responds to the blastocyst shed from the zona pellucida, leading to completion of implantation via the processes of apposition, attachment, adhesion, and encapsulation.

During an unsuccessful pregnancy, embryo implantation failure may result from embryonic factors undetectable with PGT‐A; however, aside from embryonic factors, various other factors can also intricately interfere with implantation.^1^, ^2^ In recent years, evidence for each examination and treatment approach has been reviewed, resulting in the publication of guidelines for recurrent implantation failure (RIF).^3^ Despite the lack of a consensus on test selection and treatment approaches, combination treatment based on diagnostic results from selected examinations is required for RIF, which is considered a multifactorial disease. To achieve pregnancy during the next embryo transfer (ET), this review seeks to clarify evidence‐based testing and treatment strategies after implantation failure following euploid ET.

## 2 RISK FACTORS FOR IMPLANTATION FAILURE

**FIGURE 1:**
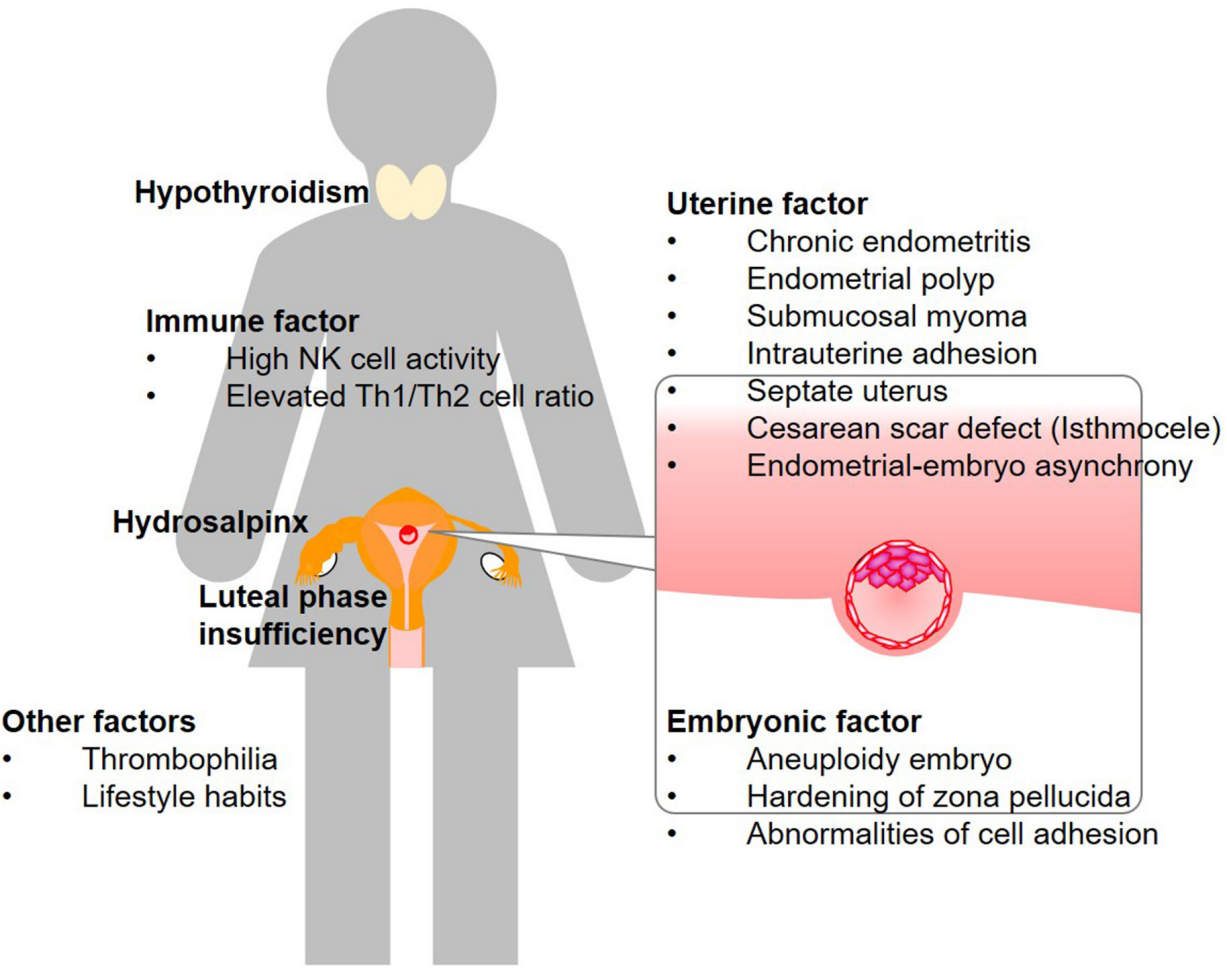
Risk factors for implantation failure. Nonembryonic risk factors for implantation failure include intrauterine abnormalities, imbalance in maternal immunity, hydrosalpinx, and luteal phase deficiency. Hypothyroidism, thrombophilia, lifestyle habits mainly increase the risk of pregnancy loss but not implantation failure. Possible embryonic risk factors undetected by PGT‐A include false‐negative PGT‐A results, hardening of the zona pellucida, and impairment in cell adhesion at implantation.

Studies have reported various inhibitory factors for embryo implantation, including uterine, immune, endocrine, microbial, hematologic, genetic, and lifestyle factors (Figure 1).^1^, ^3^ A major nonembryonic risk factor for implantation failure is an abnormal intrauterine environment, which is the site of embryo implantation. Intrauterine abnormalities include endometrial polyps, submucosal myomas, intrauterine adhesions, septate uterus, chronic endometritis (CE), and a thin endometrium. Among infertile women who had undergone a cesarean section, a cesarean scar defect may also prohibit implantation, although rarely, by impacting the inflammatory bloody fluid derived from the isthmocele.^4^ Furthermore, asynchrony between the WOI and the transferred blastocyst has been identified as another cause of unsuccessful implantation,^5^ which involves an impairment in decidual change caused by luteal phase deficiency. In the process of maternal acceptance of a semi‐allogeneic embryo, maternal immunological rejection can also be a risk factor for implantation failure. In line with this, vitamin D has been identified as an important nutrient that improves maternal immune tolerance for better embryo receptivity. Thyroid abnormalities, including hypothyroidism and Hashimoto's thyroiditis, have detrimental effects on female fertility and pregnancy.^6^, ^7^, ^8^ In patients with a hydrosalpinx, intratubal fluid with highly inflammatory mediators flow into the uterine cavity, subsequently harming the embryos and interfering with implantation.^9^, ^10^

Despite having normal results on PGT‐A with 23 pairs of chromosomes, chromosomal aberrations cannot be ruled out. Hardening of the zona pellucida^11^ and impairment in cell adhesion at implantation have also been identified as possible risk factors for implantation failure.

Studies have shown that thrombophilia, including antiphospholipid syndrome (APS), is associated with pregnancy loss. Although thrombophilia screening and anticoagulant treatment for implantation failure still remain controversial, high prevalence rates of thrombophilia have been reported in patients with a history of RIF.^12^, ^13^, ^14^ Our previous trial revealed that thrombophilia treatment was an important factor for a successful pregnancy in patients with RIF.^12^ Although thrombophilia is not a risk factor for implantation failure, its treatment is required for the prevention of pregnancy loss.

Some lifestyle habits negatively influence female fecundity.^15^ Therefore, preconception lifestyle modifications, including cessation of alcohol consumption and smoking, have been recommended for successful ART treatment.

## 3 TESTS AND TREATMENTS FOR IMPLANTATION FAILURE

Testing and treatment approaches for implantation failure are summarized in Tables 1 and 2, respectively. Our previous study found that around half of the women with RIF had two or more risk factors for implantation failure.^12^, ^16^ Therefore, the standard treatment strategy for implantation failure is to detect and adequately address risk factors of unsuccessful implantation based on the results of implantation testing. The following discussions detail each examination and treatment approach for risk factors. In this review, the risk factors that can be easily detected by screening tests for infertility, such as organic uterine abnormalities, luteal phase deficiency, thyroid disorders, and hydrosalpinx, are omitted.

**TABLE 1:** Examinations for implantation failure.

Tests for intrauterine circumstance and window of implantation *Hysteroscopy* *Endometrial CD138 immunostaining and bacterial culture with antibiotic susceptibility testing* Endometrial microbiome analysis Measurement of endometrial thickness Endometrial receptivity test for window of implantation
2 Immunological test Th1/Th2 cell level Serum NK cell activity *25‐hydroxyvitamin D*_*3*_ *(storage form of vitamin D)*
3 Examination of embryos *Preimplantation genetic testing for aneuploidy*
4 Other testing *Thrombophilia screening*

*Note*: Italics indicate evidence‐based examinations for implantation failure of euploid embryos based on systematic reviews.

**TABLE 2:** Treatments of risk factors for implantation failure.

Treatments for uterine factors *Antibiotics for CE without diseases‐causing CE* Hysteroscopic surgery or endometrial curettage for CE Estradiol treatment for thin endometrium Intrauterine PRP infusion for thin endometrium Subcutaneous or intrauterine infusion of G‐CSF administration for thin endometrium
2 Optimization of timing of embryo implantation *Frozen–thawed blastocyst transfer* Luteal phase support Personalized embryo transfer based on the result of endometrial receptivity test
3 Regulation of immune torelance Tacrolimus Adalimumab (anti‐TNF‐α) Intravenous immunoglobulin Glucocorticoid *Vitamin D supplementation for vitamin D insufficiency/deficiency*
4 Treatments for embryonic factors undetected by PGT‐A *Additional zona pellucida incision using laser‐assisted hatching* *Hyaluronan‐enriched embryo transfer medium* *Single blastocyst transfer*
5 Treatments for other factors *Lifestyle modification* ^a^ *Low‐dose aspirin* (and low‐molecular‐weight heparin) *for thrombophilia* ^a^ ^,^ ^b^

*Note*: Italics indicate evidence‐based examinations for implantation failure of euploid embryos based on systematic reviews.

Abbreviations: CE, chronic endometritis; G‐CSF, granulocyte colony‐stimulating factor; PRP, platelet‐rich plasma.

^a^ Lifestyle factors and thrombophilia are risk factors for miscarriage, but not implantation failure.

^b^ Anticoagulant therapy starting from the postimplantation period is recommended. Heparin treatment is required for only antiphospholipid syndrome in thrombophilia.

### 3.1 Tests and treatments for chronic endometritis and endometrial dysbiosis

CE is a continuous endometrial inflammatory condition involving plasmacytes across different menstrual cycles. This condition negatively influences female fertility and is present in 30%–57% of women with a history of RIF.^12^, ^17^, ^18^ CE primarily affects endometrial decidualization, altering the timing of the WOI and reducing the chances of embryo implantation.^19^, ^20^ Reports have shown that CE contributes little to female fecundity in general infertile patients^21^; however, a meta‐analysis in the patients with RIF demonstrated that recovery from CE improves both clinical pregnancy and livebirth rates (odds ratio [OR]: 4.02, 95% CI: 1.35–11.94 and OR: 6.81 95% CI: 2.08–22.24, respectively).^22^

CE is diagnosed based on the presence of plasmacytes determined through plasma cell marker CD138 immunohistochemistry of the biopsied endometrium.^23^ However, the diagnosis of CE based on the presence of 1–10 CD138‐positive plasmacytes in 1–20 nonoverlapping random stromal areas at 400‐fold magnification still remains controversial. Given that CE is mainly caused by intrauterine infection with a wide variety of microorganisms. In endometrial microbiome analysis, CE is associated with abundant bacterial microbiotas including *Bifidobacterium*, *Prevotella*, and *Ureaplasma* species.^24^, ^25^ Therefore, broad‐spectrum antibiotic therapy has been considered the gold standard for the treatment of CE.^17^, ^26^ Doxycycline administration for 2 weeks has been recommended globally as the first‐line treatment for CE due to its high cure rates (66%–93%) as previously reported.^17^, ^26^

However, not all CE cases can be attributed to endometrial infection. Various disorders, including endometrial polyps, intrauterine adhesions, hydrosalpinx, and cesarean scar defects, have been associated with incidence of CE.^27^, ^28^, ^29^, ^30^ CE cases with intrauterine abnormalities or a hydrosalpinx are usually treated through surgery without antibiotics.^27^, ^28^, ^29^ In addition, systemic antibiotic treatment for CE with endometrial polyps may decrease clinical pregnancy rates during infertility treatment after surgery.^28^ Therefore, CE caused by other organic disorders should be treated by removing the lesions first without antibiotic use.

**FIGURE 2:**
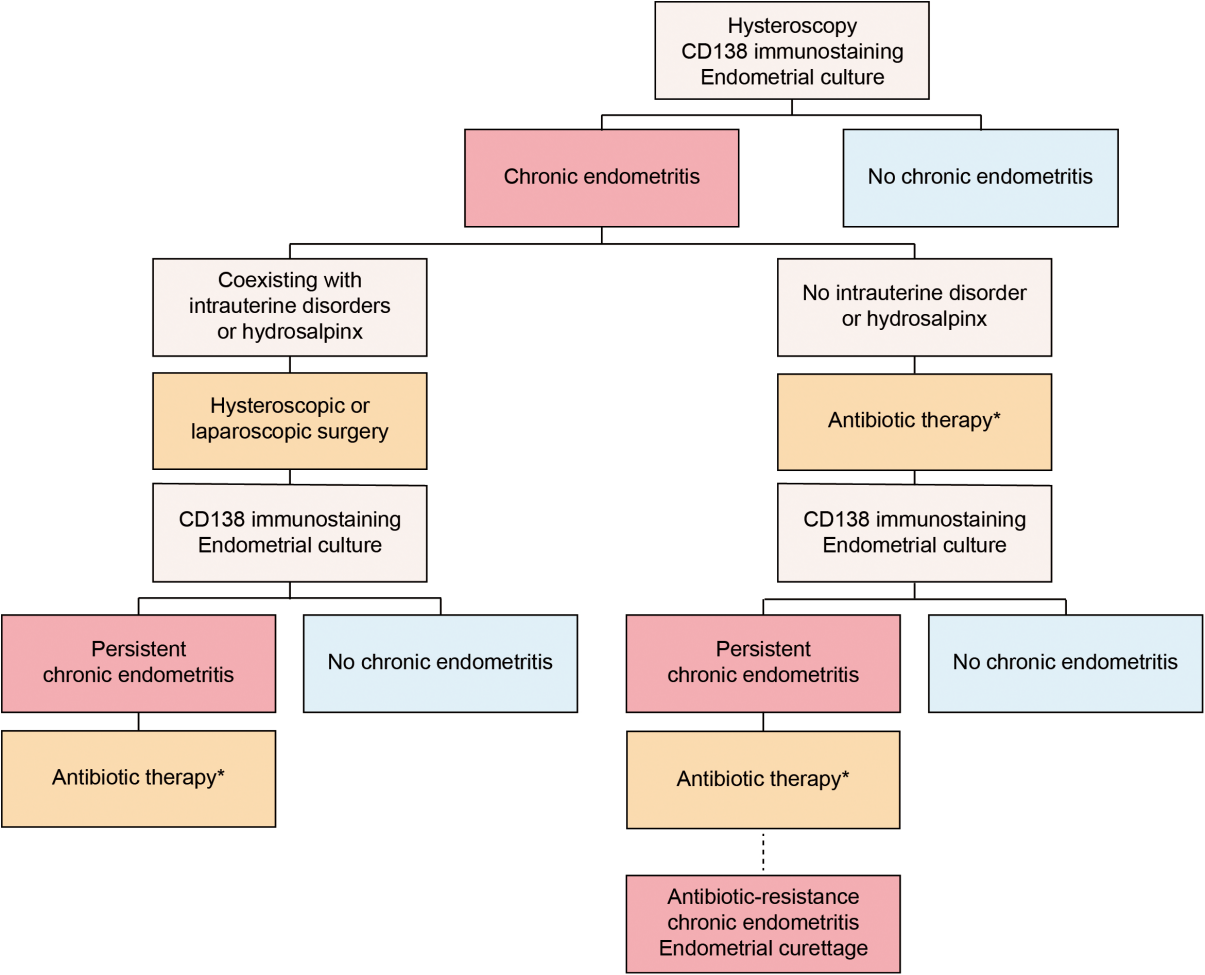
Treatment protocols of chronic endometritis. When intrauterine organic lesions or a hydrosalpinx is present, hysteroscopic or laparoscopic surgery is initially performed. Endometrial CD138 immunostaining and bacterial culture tests are then repeated without antibiotic use in the subsequent menstruation cycle after surgery. When CE is detected in patients without intrauterine lesions, oral antibiotics based on bacterial culture test or doxycycline are administered. Should CE persist after ≥2 cycles of antibiotics, the inflamed endometrium is removed via gentle curettage. *Oral bacterium‐sensitive antibiotics or doxycycline are administered, according to the results of endometrial bacterial culture tests with or without specific bacteria except for *Lactobacillus* spp. or *Bifidobacterium* spp., respectively. If CE persisted with or without specific bacteria, bacterium‐sensitive antibiotics or ciprofloxacin and metronidazole are used as second‐line therapy. Figure in Kuroda et al. *Reprod Med Biol* 2023 was modified.^16^

Previous reports have introduced the treatment protocol for CE (Figure 2).^16^ Accordingly, CE patients with intrauterine organic lesions or a hydrosalpinx should initially undergo hysteroscopic or laparoscopic surgery. Thereafter, endometrial CD138 immunostaining and bacterial culture with antibiotic susceptibility testing are performed during the next menstruation cycle without postoperative antibiotic administration. After establishing a diagnosis of CE in patients without intrauterine disorders or a hydrosalpinx, oral bacterium‐sensitive antibiotics or doxycycline are administered. Should CE persist after ≥2 antibiotic cycles, artificial removal of the inflammatory endometrium via gentle curettage using a blunt uterine curette without applying any force is recommended.^31^ In fact, our previous report found that endometrial curettage for antibiotic‐resistant CE significantly reduced the number of plasma cells, thereby increasing clinical pregnancy rates following IVF irrespective of the presence of CE.^31^ Although this is our CE treatment protocol, we believe that it needs to be further refined in the future.

In recent years, endometrial microbiome analysis, which involves sequencing the 16S ribosomal RNA in each bacterium present at the sampling site, has become a common test for intrauterine environments. Moreno et al.^32^ had been the first to demonstrate that women with endometrial *Lactobacillus*‐enriched microbial communities (*Lactobacillus* ratio ≥90%) had much better clinical pregnancy outcomes than did those with a *Lactobacillus* ratio of <90%. Conversely, Franasiak et al.^33^ reported no significant difference in endometrial microbiotas, including *Lactobacillus* spp., at the distal 5‐mm portion of the transfer catheter between patients who did and did not experience a successful pregnancy after single euploid blastocyst transfer. Likewise, Hashimoto et al.^34^ showed that dysbiosis with a low *Lactobacillus* spp. ratio did not contribute to pregnancy outcomes after ET. Furthermore, Ichiyama et al.^35^ reported no significant difference in endometrial *Lactobacillus* abundances (50%–55%) between healthy control women and patients with a history of RIF, with their rates being significantly lower than that initially reported.

Although optimal vaginal flora for pregnancy is *Lactobacillus* spp.‐dominant, endometrial microbiota species are highly diversity and limited in amount (1/100–1/10 000) compared to those in the vagina.^36^, ^37^ In addition, endometrial microbiome analysis showed only the proportion of intrauterine microbiota, not the amount of bacterium. Therefore, distinguishing between intrauterine infection and dysbiosis without pathogenic microorganisms would be impossible. Moreover, diagnostic criteria for endometrial eubiosis or dysbiosis have still not been established considering the rich diversity of endometrial microbiotas. Furthermore, microbiome analysis results show the recommended antibiotic agents that can be administered without antimicrobial susceptibility testing; however, antibiotic‐resistant CE often cannot be cured by administering the recommended antibiotics. Interestingly, a meta‐analysis of studies employing genital microbiome analysis showed that vaginal dysbiosis was associated with poor pregnancy outcomes following IVF treatment; however, the negative impact of abnormal endometrial microbiome analysis results still remain unclear.^38^

### 3.2 Treatments for cesarean scar defect

Women with cesarean scar defects and abnormal bleeding need to undergo hysteroscopic examination to confirm the site of the scar and status of surrounding tissues, with typical findings including dendritic vessels with blood retention and blackish blown spots.^4^ The current standard treatment for cesarean scar defect is hysteroscopic resection of the anterior and posterior corners of the scar defect and coagulation of the bottom of the scar. Should the residual myometrial scar be exceedingly thin (<3 mm), laparoscopic removal and resuturing of the scar should be considered for the prevention of uterine rupture during pregnancy.^4^, ^39^

### 3.3 Diagnosis and treatments for thin endometrium

Endometrial thickness has been identified as a prognostic factor for pregnancy outcomes following IVF treatment.^40^ However, no consensus has yet been established regarding what can be considered a “thin” endometrium on ultrasonography. Data from Canada on >14 000 frozen–thawed blastocyst transfer cycles showed that clinical pregnancy rates in women with endometrial thickness ≥8.0, 7.0–7.9, 6.0–6.9, and <6.0 mm were 40.6%, 40.4%, 35.4%, and 29.4%, respectively.^41^ Therefore, an endometrial thickness of <7 mm could be considered to be involved in the decline in pregnancy rates after frozen–thawed blastocyst transfer. However, a recent retrospective study on 959 single euploid ET cycles found that endometrial thickness was not associated with pregnancy outcomes.^42^ As such, when competent blastocysts are transferred, a thin endometrium may not be a cause for concern.

Should a patient desire additional therapy for a thin endometrium, administering a sufficient amount of estradiol is still the primary treatment approach.^43^ However, for patients with a thin endometrium complicated by intrauterine adhesions, hysteroscopic adhesiolysis is needed. Regenerative medicine can be used as further treatment for endometrial damage. Endometrial stem cells play a significant role in cyclic endometrial regeneration, including proliferation, decidualization, and shedding. Indeed, a systematic review and meta‐analysis on the use of granulocyte colony‐stimulating factor (G‐CSF) found that both subcutaneous and intrauterine infusion of G‐CSF improved clinical pregnancy rates, although systemic administration was more effective (subcutaneous RR: 1.73, 95% CI: 1.33–2.23; infusion RR: 1.39, 95% CI: 1.09–1.78).^44^ Moreover, in vivo and in vitro studies showed that intrauterine platelet‐rich plasma (PRP) infusion also had endometrial proliferative effects.^45^, ^46^ Meanwhile, another meta‐analysis showed that PRP infusion significantly increased endometrial thickness and improved clinical pregnancy rates in women with thin endometrium or RIF (thin endometrium, RR: 1.79, 95% CI: 1.37–2.32; RIF, RR: 1.73, 95% CI: 1.24–2.41).^47^ Overall, evidence suggests that regenerative medicine has potential therapeutic efficacy for RIF with an abnormal intrauterine environment.

### 3.4 Optimization of timing of embryo implantation

A successful pregnancy requires synchronization between a competent embryo and timing of the WOI.^5^ An endometrial receptivity test, namely the endometrial receptivity analysis (ERA), is a tool used to identify the individual WOI based on endometrial gene expression identified from biopsied endometrium during blastocyst transfer.^48^ The results of this test allow for personalized ET at each WOI timing. Although ERA testing has been widely used globally to test for RIF, a meta‐analysis found no significant difference in both clinical and ongoing or livebirth rates between women who did and did not undergo ERA (OR: 1.14, 95% CI: 0.70–1.85 and OR: 1.38, 95% CI: 0.79–2.41, respectively).^49^ Moreover, subgroup analyses based on the number of previous ET cycles demonstrated that ERA exerted no beneficial effects for implantation failure.^49^ Furthermore, a meta‐analysis focusing on ET using euploid embryos found no significant difference between pregnancy outcomes among women who did and did not undergo ERA (OR: 0.89, 95% CI: 0.59–1.35).^50^ Based on current evidence, we can surmise that endometrial receptivity test is not recommended after failed euploid ET.

The presence of CE has been associated with “nonreceptive” results on ERA testing, suggesting that CE shifts or eliminates the WOI.^20^ Hence, comparing pregnancy outcomes after ET between women confirmed to have no CE who did and did not undergo endometrial receptivity testing can reveal the true effectiveness of ERA.

### 3.5 Regulation of immune tolerance

A successful pregnancy requires maternal immune tolerance to the semi‐allogeneic embryo. Therefore, excessive immune response has been widely known to interfere with embryo implantation. The most abundant immunologically competent cells in the process of implantation and pregnancy are natural killer (NK) cells, lymphocytes helper‐T (Th) cells, and macrophages.

The impact of peripheral or uterine NK cells on embryo implantation remains uncertain.^51^ Nonetheless, a meta‐analysis showed that several studies found a relationship between the proportion of cytotoxic peripheral NK cells (CD56^+^) and female fecundity, despite the heterogeneity among the studies, thereby suggesting their negative impact on implantation and miscarriage.^52^

Unlike peripheral NK cells, uterine NK cells (CD56^bright^) have little cytotoxicity and play an important role in the establishment of early pregnancy by inducing decidual angiogenesis, spiral arterial remodeling, and trophoblast invasion.^53^ A meta‐analysis found that although women with RIF had significantly more uterine NK cells than did controls, no relationship was observed between uterine NK cell count and pregnancy outcomes in women with RIF.^54^ Glucocorticoids, which are immunosuppressive agents, can suppress the increase in NK cells via glucocorticoid receptors as surface nuclear receptors^55^; however, the benefits of glucocorticoids on implantation failure has yet to be verified.^56^ Moreover, its excessive anti‐inflammatory effects may inhibit the important transient increase in proinflammatory cytokines at implantation^57^; therefore, unnecessary glucocorticoid treatment for implantation failure is not recommended.

Helper‐T cells associated with pregnancy include interleukin (IL)‐2‐, interferon‐γ‐, and tumor necrosis factor‐alpha‐producing Th1 cells; IL‐4‐, IL‐5‐, and IL‐10‐producing Th2 cells; IL17‐producing Th17 cells; and regulatory T (Treg) cells. Balancing Th1 or Th17 and Th2 cell levels in favor of Th2 cells is important for a successful pregnancy.^58^, ^59^ Treg cells play an important role in controlling maternal tolerance to paternal alloantigen by regulating Th cell balance.^59^ An imbalance in Th1/Th2/Th17 cell levels with a bias toward elevated Th1 or Th17 cell levels was involved in immune rejection of embryos or fetuses, promoting implantation failures and miscarriages.^59^ Studies on the roles of Th cells during pregnancy have frequently reported a relationship between elevated Th1/Th2 cell ratio and implantation failure.^60^, ^61^, ^62^ In addition, reports have revealed improved pregnancy outcomes after immunomodulation therapy, such as adalimumab, intravenous immunoglobulins, and tacrolimus, in patients with RIF who have aberrantly high Th1/Th2 cell ratios.^62^, ^63^, ^64^ However, no meta‐analysis has yet been available due to the wide variety of Th cell‐related cytokines. Evidence on the impact of the Th1/Th2 cell ratio and its treatments on pregnancy outcomes has not been established.

Our previous report, which compared fertile and infertile women with and without a history of RIF, showed no significant difference in the Th1/Th2 cell ratio between fertile and infertile women with ≤3 ET cycles; however, significantly higher ratios were recognized among women with ≥4 cycles.^61^ One observational study on infertile women without previous IVF treatment found no relationship between the Th1/Th2 cell ratio prior to IVF and subsequent IVF outcomes without immunomodulation therapy.^61^ Taken together, evidence shows that women with aberrantly high Th1/Th2 cell ratios and a history of ≥4 implantation failure ET cycles should be the therapeutic target for immunotherapy; however, immunotherapy should not be recommended for women without RIF.

Macrophages including proinflammatory M1 and anti‐inflammatory M2 subtypes also play important roles in immune tolerance for successful pregnancy.^65^ Activated M1 macrophages produce inflammatory cytokines, shifting toward Th1 immune response, and M2 macrophages have immunosuppressive effects alternatively, leading to promoting Th2 response.^66^ Therefore, an imbalance of M1 and M2 macrophages at the maternal‐fetal interface is associated with recurrent reproductive failure.^67^, ^68^ The imbalance of M1/M2 macrophages is also the potential target for immunotherapy in the future, yet the evidence is insufficient.

Considering that Vitamin D exerts immunomodulatory effects, such as suppressing optimally high NK, Th1, and Th17 cell activities and promoting Th2 and Treg cell levels,^69^ it has been considered important for pregnancy. Furthermore, vitamin D deficiency is generally asymptomatic and often underestimated in health^70^; nevertheless, it has been associated with infertility and pregnancy complications, including miscarriages and preeclampsia.^71^, ^72^, ^73^ In fact, our previous study found that 87.3% of Japanese infertile women had low levels of the storage form of vitamin D, namely 25‐hydroxyvitamin D_3_ (25OHVD <30 ng/mL), which was associated with elevated Th1/Th2 cell ratios.^74^ Vitamin D replacement can suppress elevated Th1 cell levels in blood and the local endometrium.^74^ In particular, women with 25OHVD levels ≥30 ng/mL after supplementation showed a significant decrease in their previously high Th1 levels and Th1/Th2 cell ratios. Although the European Society of Human Reproduction and Embryology (ESHRE) guideline for RIF does not recommend vitamin D testing and replacement given the lack of established evidence,^3^ a recent systematic review and meta‐analysis demonstrated that clinical pregnancy rates in IVF treatment were increased by 2.06‐fold after vitamin D replacement in women with 25OHVD levels <30 ng/mL (95% CI: 1.32–3.22).^75^ Among such women, those who received daily supplementation for >30 days had significantly better clinical pregnancy rates, compared to weekly or longer intervals and/or <30 days. In our previous report, vitamin D replacement at 25 μg/day for 3 months in patients with 25OHVD levels <30 ng/mL promoted sufficient levels in only half of them.^74^ Therefore, vitamin D supplementation at 25 and 50 μg/day is recommend to achieve 25OHVD levels of ≥20 ng/mL and between 20 and 30 ng/mL, respectively. Through this protocol, 80%–90% of women achieved a reduction in their Th1/Th2 cell ratios, half of whom attained normalized levels.^12^, ^16^, ^76^ Vitamin D supplementation is safe in doses up to 100 μg/day and has beneficial effects on not only infertility but also obstetrical and fetal complications.^77^

### 3.6 Treatments for embryonic factors undetected by PGT‐A

Euploidy results following PGT‐A have a potential risk for being false negative, with euploid cells in biopsied trophectoderm and aneuploid cells in nonbiopsied inner cell mass. Moreover, PGT‐A cannot identify microdeletions and duplications as well as de novo pathogenic mutations.^78^ A previous retrospective cohort study involving 4429 women who underwent up to three consecutive frozen–thawed euploid ETs showed implantation rates of 69.9%, 59.8%, and 60.3% after the first, second, and third ET, respectively.^79^ Therefore, implantation failure can be presumably attributed to embryonic factors undetected by PGT‐A, with simply repeating ET being one option. One meta‐analysis showed that morphologically and developmentally poor blastocysts can cause lower pregnancy rates even in euploid embryos (Grade < BB of Gardner's classification, OR: 0.40, 95% CI: 0.24–0.67; Day 6–7 blastocysts, OR: 0.56, 95% CI: 0.49–0.63).^50^ Furthermore, zona pellucida drilling at day 3 cleavage stage embryos and blastocyst biopsy, double vitrification, and fresh ET have also been found to negatively impact clinical pregnancy rates in euploid ET.^50^

Hardening of the zona pellucida is involved in in vivo aging, prolonged in vitro culture, or embryo freezing.^11^ Assisted hatching slightly improves clinical pregnancy rates (OR: 1.20, 95% CI: 1.09–1.33)^80^ but may increase the risk of multiple pregnancy^81^; hence, the ability of this technique to improve livebirth rates still remains controversial.^80^ Notably, using a small hole size during assisted hatching has been associated with poor pregnancy outcomes^82^ and increased risk for zygotic splitting via herniating of the blastocyst at hatching.^83^ Therefore, when the hole is drilled in zona at biopsy is small, additional zona pellucida incision through laser‐assisted hatching is recommended after thawing.

Impairment of cell adhesion at implantation is another possible risk factor for implantation failure. Hyaluronan‐enriched transfer medium promotes embryo attachment to the endometrium. Indeed, evidence shows that the use of hyaluronan‐enriched medium during ET slightly improves clinical pregnancy rates (RR: 1.16, 95% CI: 1.09–1.23).^84^ Moreover, one study showed that hyaluronan‐enriched medium may support embryo implantation after transfer using morphologically poor‐quality euploid blastocysts.^85^

Some reports have revealed that aneuploidy and/or poor‐quality embryos adversely influence endometrial receptivity of the decidual endometrium.^86^, ^87^ In fact, a systematic review and meta‐analysis found no significant difference of clinical pregnancy rates after frozen–thawed ET using a single good‐quality embryo and double poor‐ and good‐quality embryos (RR: 0.97, 95% CI: 0.83–1.15).^88^ Although no study has yet investigated implantation rates per embryo, a single good‐quality ET could presumably promote higher implantation rates. Furthermore, another study showed that cumulative livebirth rates were higher after two consecutive cycles of single blastocyst transfer than after one cycle of double blastocyst transfer (OR: 1.33, 95% CI: 1.29–1.38).^89^ Furthermore, double ET increased the risks of multiple pregnancy and maternal and fetal complications during pregnancy.^88^, ^89^ Taken together, evidence suggest that a single ET is recommended to maximize the fertile potential of a euploid embryo.

### 3.7 Modification of lifestyle

Various lifestyle behaviors negatively impact pregnancy after IVF treatment.^15^ However, the effectiveness of lifestyle modification in improving pregnancy outcomes still remains controversial. Nonetheless, evidence shows that maternal and/or paternal smoking of >10–20 cigarettes per day, alcohol drinking of >2 times per week, caffeine intake of >2–3 cups of coffee per day, and obesity (BMI >30) are lifestyle factors that increase the risk of pregnancy loss by 1.5‐ to 2‐fold.^90^ Thus, evaluation and modification of lifestyle habits, such as discontinuing smoking and caffeine and alcohol consumption during pregnancy and engaging in exercise and calory restriction for obesity, is recommended in ESHRE guideline for RIF.^3^ Maternal phycological stress has also been associated with infertility and pregnancy loss.^91^, ^92^ Therefore, counseling is recommended to reduce maternal stress and anxiety as necessary.

**FIGURE 3:**
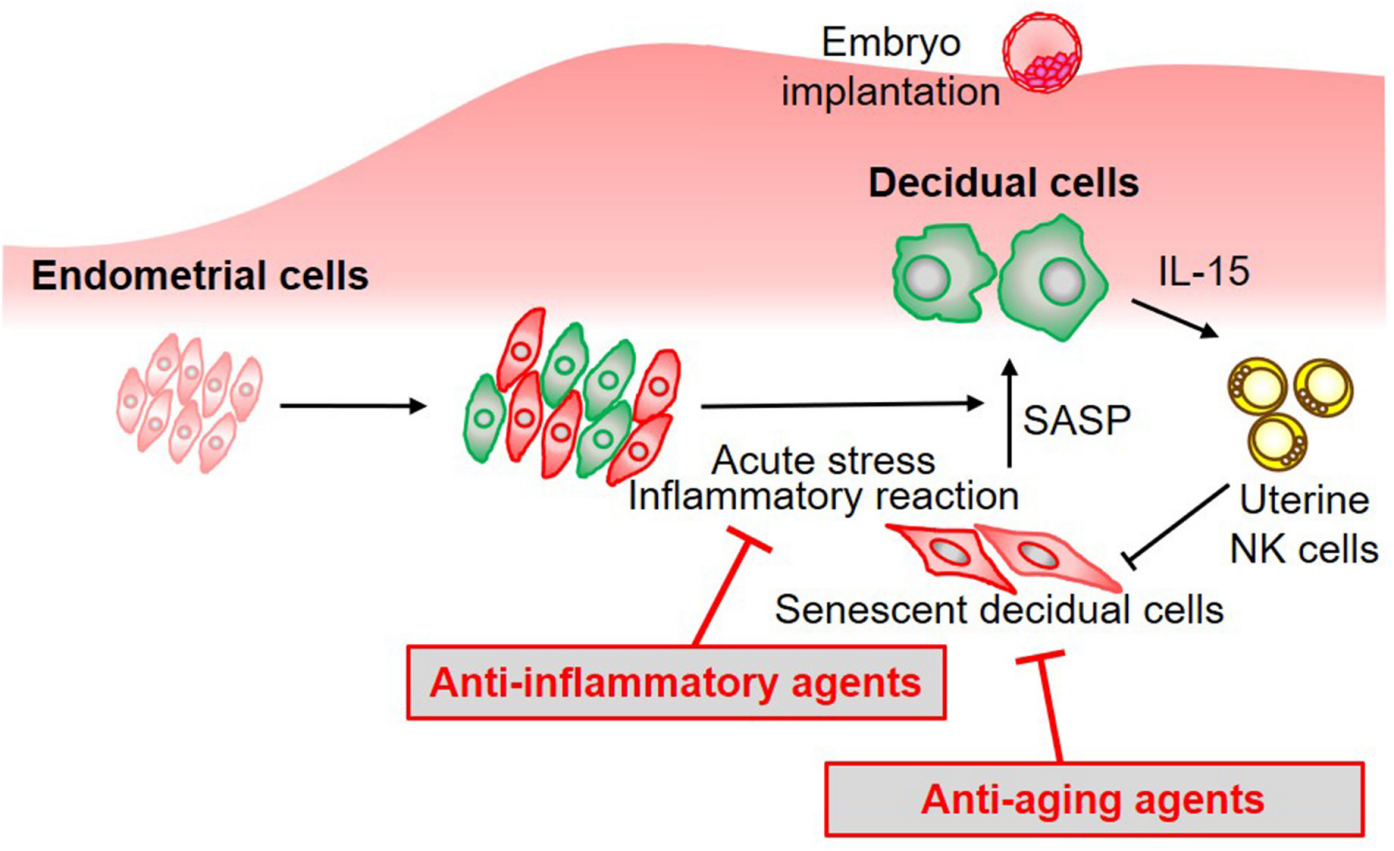
Drugs and supplements that may inhibit decidualization and implantation. During endometrial decidualization, stress‐resistant decidual endometrial cells emerge due to an acute stress response, whereas some decidual cells burdened by replication stress transform into senescent decidual cells. Given that uterine NK cells eliminate senescent decidual cells, endometrial cells transform into decidual cells. At implantation, the decidual endometrium modulates a local inflammatory reaction that allow trophoblast invasion by secreting proinflammatory cytokines. Throughout this multistep process, anti‐aging or anti‐inflammatory drugs or supplements may inhibit decidualization and implantation.

Patients who suffer from repeated IVF failures often self‐manage their reproductive care using multiple medications and supplements without robust evidence. During the decidual transformation of the endometrium, stress‐resistant decidual endometrial cells emerge through an acute stress response, whereas some decidual cells burdened by replication stress transform into senescent decidual cells (Figure 3). Given that uterine NK cells eliminate senescent decidual cells, endometrial cells transform into morphologically and functionally different decidual cells.^93^, ^94^ At implantation, the decidual endometrium modulates a local inflammatory reaction that allows trophoblast invasion by secreting proinflammatory cytokines and prostaglandins.^95^, ^96^ Throughout this multistep process, senolytics or anti‐inflammatory drugs, such as antiaging supplements, antipyretics, and analgesics, could potentially inhibit decidualization and embryo implantation.^97^ In fact, studies have shown that the antiaging agents resveratrol and rapamycin prohibit endometrial decidualization.^93^, ^98^ Although systemic or local excessive inflammatory responses have been associated with reproductive failure, the routine use of anti‐inflammatory drugs during IVF treatment does not improve pregnancy outcomes.^99^, ^100^, ^101^ Unnecessary medications and supplements with potential negative effects on implantation should be discontinued during ET cycles.

### 3.8 Thrombophilia screening and anticoagulant therapy

One possible mechanism by which thrombophilia causes implantation failure is local microvascular thrombosis and impaired blood flow of decidual or chorionic vessels at the site of embryo implantation.^102^ Given reports showing a high prevalence rate of thrombophilia (19%–69%), including APS, in patients with a history of RIF,^12^, ^13^, ^14^ thrombophilia screening and prevention of miscarriage are needed for patients with RIF. However, a large‐scale study including infertile patients who underwent IVF treatment found that thrombophilia was not associated with the number of IVF cycles and implantation failure. Instead, the study showed that women with activated protein C resistance and/or lupus anticoagulant factor V Leiden had better pregnancy outcomes than did those without thrombophilia.^103^ Furthermore, a previous RCT on anticoagulant treatment for RIF revealed that low‐molecular‐weight heparin and low‐dose aspirin did not increase clinical pregnancy rates in women with antiphospholipid antibody or antinuclear antibody (6.8% and 8.5% in the treatment and placebo group, respectively).^104^ Similarly, a meta‐analysis showed that low‐molecular‐weight heparin treatment in RIF women without APS reduced miscarriage rates (RR: 0.22, 95% CI: 0.06–0.78) and increased livebirth rates (RR: 1.79, 95% CI: 1.10–2.90) but did not improve implantation rates (RR: 1.73, 95% CI: 0.98–3.03).^105^ The prevalence of inherited or acquired thrombophilia, except for APS, is high in women with RIF^12^, ^13^, ^14^; therefore, anticoagulant treatment could prevent pregnancy loss but not improve embryo implantation. Given that heparin has some serious side effects, including bleeding, allergic reactions, osteoporosis, and heparin‐induced thrombocytopenia^106^, ^107^; unnecessary use of heparin should be avoided. Meanwhile, studies have shown that the routine use of low‐dose aspirin during IVF treatment does not support embryo implantation.^101^, ^108^ Aspirin and heparin are usually administered after confirming pregnancy; therefore, anticoagulant therapy should be started during the postimplantation period, not before implantation, when needed.

## 4 TREATMENT STRATEGY FOR IMPLANTATION FAILURE AFTER A EUPLOID EMBRYO FAILS TO IMPLANT

According to a large number of clinical studies and systematic reviews, evidence‐based etiologies for euploid embryo implantation failure include (1) CE, (2) vitamin D insufficiency/deficiency, and (3) morphologically or developmentally poor embryo quality. Furthermore, risk factors for miscarriage, but not implantation failure, include (4) lifestyle factors, such as smoking, caffeine and alcohol consumption, obesity, and stress, and (5) thrombophilia.

The cornerstone for the treatment of implantation failure is to identify and address the causes of the unsuccessful pregnancy. Before subsequent euploid ET, the following additional examinations are recommended: (1) hysteroscopy, (2) endometrial CD138 immunohistochemistry and bacterial culture with antibiotic susceptibility testing, (3) serum 25OHVD level testing, and (4) thrombophilia screening (Table 1).

The following treatments are recommended based on diagnostic findings: (1) antibiotics for CE without diseases‐causing CE, (2) vitamin D replacement, (3) lifestyle modification including discontinuation of unnecessary medications and supplements, and (4) low‐dose aspirin starting from the postimplantation period for thrombophilia (Table 2). In addition, frozen–thawed single euploid blastocyst transfer with an additional zona pellucida incision via laser‐assisted hatching and use of hyaluronan‐enriched medium are also recommended.

When any risk factors for implantation failure are not detected, the embryonic factors undetected by PGT‐A may have accidentally prevented conception. Therefore, additional treatments for implantation failure may not be needed. However, further treatments, such as estradiol treatment and/or regenerative medicine for a thin endometrium, endometrial receptivity test and personalized ET, and immunotherapy for high Th1/Th2 cell ratios, can be considered. In particular, immune factors have been considered the missing piece in determining the risk factors for implantation failure due to insufficient evidence. Patients who have a history of RIF and aberrantly high Th1/Th2 cell ratios, which remain uncontrolled after vitamin D supplementation, may benefit from tacrolimus therapy.^12^, ^62^

The OPtimization of Thyroid function, Thrombophilia, IMmunity and Uterine Milieu (OPTIMUM) treatment strategy, which combines remedies for RIF and recurrent pregnancy loss (RPL),^12^, ^76^ can detect risk factors for implantation failure and pregnancy loss using minimum and inexpensive tests and treated the identified factors. Studies have shown that most women aged <40 years with a history of RIF and/or RPL were able to achieve childbearing after the OPTIMUM.^12^, ^16^, ^76^ The OPTIMUM has been introduced in ESHRE guideline for RIF.^3^ Hence, a combination protocol, such as the OPTIMUM, may promote a successful pregnancy at next ET among patients who experienced euploid embryo implantation failure.

## 5 CONCLUSION

Patients who experience implantation failure after euploid ET lose hope in ever achieving future pregnancy. Implantation failure has been associated with a wide variety of risk factors, with existing evidence recommending combination protocols based on diagnostic findings. Moreover, a single euploid blastocyst transfer using assisted hatching and hyaluronan‐enriched medium may support embryo implantation. However, a euploid blastocyst may fail to implant due to embryonic factors that remain undetected by PGT‐A. Thus, simple, inexpensive, and evidence‐based tests and treatments should be selected.

## CONFLICT OF INTEREST STATEMENT

The authors declare no conflict of interest.

## ETHICS STATEMENT

All procedures followed were in accordance with the ethical standards of the responsible committee on human experimentation and with the Helsinki Declaration of 1964 and its later amendments. All recruited women provided written informed consent.

## ANIMAL STUDIES

This article does not contain any study with animal participants that have been performed by any of the authors.

## Data Availability

The data that support the findings of this study are available on request from the corresponding author. The data are not publicly available due to privacy or ethical restrictions.
